# Influence of extreme flows on habitat and fish assemblage structure in groundwater-dominated systems

**DOI:** 10.7717/peerj.21092

**Published:** 2026-04-27

**Authors:** Joshua D. Tivin, Timothy H. Bonner

**Affiliations:** 1 Department of Biology, Texas State University, San Marcos, TX, United States; 2 The Meadows Center for Water and the Environment, San Marcos, TX, United States

**Keywords:** Drought, Flood, Spring systems, San marcos river, Comal river, Hydrologically stable systems, Texas, Fish community, Disturbance ecology

## Abstract

**Background:**

Periods of extreme flow (*i.e*., drought and flood) structure aquatic communities, but their effects in hydrologically stable, groundwater-dominated systems (*e.g*., karst springs) remain poorly understood. These spring systems are often viewed as refugia for endemic fishes. As such, the effects of drought and flooding are thought to be more pronounced than in hydrologically variable systems with the potential to lead to extirpations of endemic fishes.

**Methods:**

We analyzed a 9-year dataset (2014–2022) from three reaches of the San Marcos River and four reaches of the Comal River, Texas, to evaluate how a major flood and a severe drought influenced habitat structure and fish assemblages among wadeable and non-wadeable areas. Habitat variables were surveyed alongside standardized fish sampling, and fishes were grouped into habitat usage guilds for analysis.

**Results:**

Effects of extreme flow periods were similar to the effects reported for hydrologically variable systems in wadeable areas but not in non-wadeable areas. Among wadeable areas, an increase in the amount of algae was detected during drought, but changes to substrates and vegetation coverage because of scouring were not detected following a flood. Also, abundances of pelagic generalist fishes (*e.g*., *Lepomis* sp., *Herichthys cyanoguttatus*) and one species of a pelagic specialist fish (*i.e*., *Astyanax argentatus*) decreased following a flood. Unexpectedly, abundances of benthic guild fishes (*i.e*., *Etheostoma* sp.) increased during drought and abundances of one species of pelagic specialist fish (*Dionda nigrotaeniata*) decreased following a flood.

**Conclusions:**

Effects of extreme flow periods on habitat structure and fish assemblages in hydrologically stable systems were similar to, or unexpectedly less pronounced, than the effects of flow periods on hydrologically variable systems. These patterns reveal vulnerabilities among generalist species during high flows and drought-associated increases for benthic taxa. These results support ecological theory that groundwater-dominated systems provide resistance to climatic extremes but remain susceptible to community restructuring, with implications for conservation under future climate variability and groundwater extraction.

## Introduction

Extreme flows in the form of major floods and severe droughts are primary drivers in structuring aquatic habitats and communities (Pearsons, Li & Lamberti, 1992). Major floods, defined as any high flow event that affects aquatic biota (Matthews, 1986), erode stream channels, alter substrate composition, remove vegetation, and displace aquatic organisms (Harrell, 1978; Matthews, 1986; Hastie et al., 2001; Sotola et al., 2021). Droughts, defined as periods of unpredictable low flow events that affect aquatic biota (Humphries & Baldwin, 2003), degrade water quality, increase algal blooms, and increase aquatic biota mortalities (Bunn & Arthington, 2002; Dahm et al., 2003; Magoulick & Kobza, 2003; Rolls, Leigh & Sheldon, 2012). Predator-prey interactions and abiotic stressors (*e.g*., physiochemical changes, habitat alterations) are also intensified during drought conditions and can result in assemblage structural changes (Franssen et al., 2006). While extreme flows directly alter aquatic biota through displacement or increased mortality rates, floods and droughts can produce indirect and prolonged effects by altering habitat structure of river reaches, and in turn, delaying the recovery of aquatic biota by reducing niche (Hakala & Hartman, 2004) and resource availability (Walters & Post, 2011; Rolls, Leigh & Sheldon, 2012; portions of this text were previously published as part of a preprint: Tivin, 2023).

Habitat structure within lotic systems strongly influences the abundance and diversity of aquatic biota (Gorman & Karr, 1978; Willis, Winemiller & Lopez-Fernandez, 2004). Greater habitat structure (*e.g*., woody debris, vegetation cover, substrate diversity) in three-dimensional space is positively correlated with fish diversity and abundance and can also be attributed to habitat support for a diversity of specialized functional traits and water-column niches (Tramer & Rodgers, 1973; Gorman & Karr, 1978; Turgeon, Turpin & Gregory-Eaves, 2019). Within biotic communities, macroinvertebrates and fishes are more concentrated (*e.g*., species packing, niche partitioning) in areas of greater habitat structure and therefore greater niche and resource availability (Rolls, Leigh & Sheldon, 2012). Stream flow, including extreme flows, is a major determinant of habitat structure and, therefore, a determinant of biotic composition, trophic structure, and carrying capacity within aquatic communities (Poff et al., 1997; Bunn & Arthington, 2002; Magoulick & Kobza, 2003; Olden & Kennard, 2010; Rolls, Leigh & Sheldon, 2012).

The predictability, intensity, and frequency of flows (*e.g*., seasonal drying *vs*. supra-seasonal drought, snowmelt runoff *vs*. flash flood) describes fundamental axes of a system’s natural flow regime that influences fish assemblage responses (Poff & Allan, 1995; Poff et al., 1997; Lynch, Leasure & Magoulick, 2018; Magoulick et al., 2021; Fox & Magoulick, 2022). Fishes inhabiting variable systems often exhibit drought adaptations (*e.g*., dissolved oxygen tolerance, generalist feeding) and flood adaptations (*e.g*., hydrodynamic morphology, opportunistic life history) that increase species resilience and recovery (Poff & Allan, 1995; Magoulick & Kobza, 2003; McManamay & Frimpong, 2015; Perkin et al., 2019). The role of extreme flows has been explored in hydrologically variable systems, indicating that extreme low flows alter habitat structure and community response through habitat disconnectivity, dispersal limitations, and local extinctions (Magoulick & Kobza, 2003; Rolls, Leigh & Sheldon, 2012; Magoulick et al., 2021), whereas high flows scour substrates, uproot vegetation, and displace individuals (Lake, 2000, Rolls, Leigh & Sheldon, 2012).

Interrelationships among stream flow extremes, habitat structure, and stream fish assemblages are established in streams and rivers with flows dominated by surface run-off (Ross & Baker, 1983; Willis, Winemiller & Lopez-Fernandez, 2004; Magoulick et al., 2021; Gonçalves-Silva et al., 2022). Less known are the effects of unpredictable stream flow extremes on habitat structure and fish assemblages within hydrologically stable aquatic systems, such as perennial spring systems, with surface flows dominated by groundwater discharge. Hydrologically stable aquatic systems are often considered as hydrologic refugia (Keppel et al., 2015) and evolutionary refugia (Davis et al., 2013; Craig et al., 2016), where local aquatic environments are decoupled from surrounding climates (*e.g*., spring systems in arid and semi-arid regions) and typically support endemic fauna, including several species of conservation concern (BIO-WEST, Inc., 2024). The effects of extreme flow events on habitat structure and existing fish assemblages have thought to be more pronounced in hydrologically stable systems, leading historical claims that minor alterations in habitat (Hubbs & Peden, 1969) and unpredictable flow extremes (Schenck & Whiteside, 1976) could lead to extirpation of endemic fishes (*e.g*., San Marcos Gambusia *Gambusia georgei*; Fountain Darter *Etheostoma fonticola*). More recently, flow-ecology relationships were quantified in groundwater flashy streams of the Ozark Highlands, where high flow metrics were more important in structuring the community than low flow metrics (Lynch, Leasure & Magoulick, 2018).

The purpose of this study was to assess how an unpredictable flash flood and heightened summer drought influenced the habitat structure and fish assemblages of two groundwater-dominated systems using a 9-year dataset. The objectives of this study were to: (1) assess the effects of extreme flows on habitat structure (*e.g*., vegetation cover, water depth, substrate composition), and (2) quantify flow extreme—fish assemblage relationships using habitat usage guilds (*i.e*., generalist *vs*. specialists; water-column associations) in wadeable areas (<1.6 m in depth) and non-wadeable areas (>1.6 m in depth with some exceptions during periods of low flow). Based on established flow—habitat relationships (Poff & Allan, 1995; Poff et al., 1997; Magoulick & Kobza, 2003; De Wilde et al., 2014), we hypothesized that flooding would scour substrates, uproot vegetation, and increase water depths (Pearsons, Li & Lamberti, 1992; Poole et al., 2022), drought would increase siltation, decrease water depths, and promote algal growth (Bornette & Puijalon, 2011; Rolls, Leigh & Sheldon, 2012; Magoulick et al., 2021), and the effects would be more pronounced in wadeable areas than in non-wadeable areas because wadeable areas are more susceptible to drying during drought (Magoulick & Kobza, 2003; Magoulick et al., 2021) and, with lower hydraulic retention, more susceptible to flood pulse energy (Wohl, 2020). Additionally, we expected fish responses would be influenced directly (*e.g*., downstream displacement) and indirectly (*e.g*., parallel habitat changes) by extreme flows, more so in wadeable areas than in non-wadeable areas. Specifically, we hypothesize that abundance of surface fishes (*i.e*., *Gambusia sp*.) would decrease following a flood event (Meffe, 1984), abundances of pelagic generalists (*e.g*., Centrarchidae) would decrease during drought and following a flood (Chang et al., 2024), and abundances of pelagic specialists (*e.g*., Leuciscidae) would be unaffected by either drought or flood (Carlson & Lauder, 2011; Schlechte & Fleming, 2015). For the benthic habitat usage guild, which consisted primarily of the federally listed Fountain Darter, our expectations were based on possible explanations from Schenck & Whiteside (1976) for the extirpation of Fountain Darters in the Comal River, where extirpation was related to extreme drought conditions in the 1950s or a large flood event in the 1970s. Consequently, we expected the abundances of benthic habitat usage guild to decrease during drought and following a flood event.

## Materials and Methods

### Study area

This study was conducted in two spring-fed rivers of the Edwards Plateau, a karst region in central Texas characterized by extensive groundwater storage in the Edwards Aquifer. San Marcos Springs and Comal Springs provide the primary baseflow sources for the upper San Marcos and Comal rivers, respectively, producing hydrologically stable, low-gradient systems with minimal flow variability (CV of mean daily discharge: 0.7 in the upper San Marcos River and 1.1 in the Comal River between 1995–2022; U.S. Geological Survey, 2024). San Marcos and Comal springs supply high magnitude, consistent baseflows (4.9 m^3^/s in the San Marcos River; 8.5 m^3^/s in the Comal River), which buffer both systems from regional precipitation patterns and decouple discharge from local drought conditions (Craig et al., 2016). Their small catchment areas further limit flashy high-flow events, resulting in rivers with predictable discharge regimes that contrast sharply with runoff-dominated systems (Leasure, Magoulick & Longing, 2016, Fig. 1). Seven reaches within 4.5 km of spring outflows were selected for this study. Three reaches in the upper San Marcos River (Hays County, Texas) and four reaches in the Comal River (Comal County, Texas) were sampled from May 2014 through November 2022. In the San Marcos River, reaches from upstream to downstream were (1) Spring Lake, (2) upper San Marcos River, and (3) lower San Marcos River. In the Comal River, reaches from upstream to downstream were (1) Upper Spring Run, (2) Landa Lake, where flow is manipulated to flow into (3) Old Channel, and (4) New Channel (Table 1; Fig. 2). Reaches in both rivers were separated by low-head dams or occurred in distinct spring channels, creating hydrologically isolated units. These barriers prevent routine fish movement under normal baseflows (Porto, McLaughlin & Noakes, 1999), with dispersal occurring only during rare high-flow pulses (Lucas & Baras, 2008). Because reaches were separated by ~0.64–1.33 rkm and were largely disconnected with minimal exchange of individuals under typical conditions, spatial dependence was not expected. Thus, reaches were treated as independent observational units in all subsequent analyses. Sampling periods were primarily in the Fall (October and November) and Spring (April and May) with an occasional sampling event during periods of low flows as specified by the Edwards Aquifer Habitat Conservation Plan for biomonitoring (ICF, 2022). Numbers of sampling periods were 20 in the San Marcos River (*N* = 9 in Fall, *N* = 9 in Spring, and *N* = 2 in Summer) and 22 in the Comal River (*N* = 9 in Fall, *N* = 10 in Spring, and *N* = 3 in summer).

**Figure 1:**
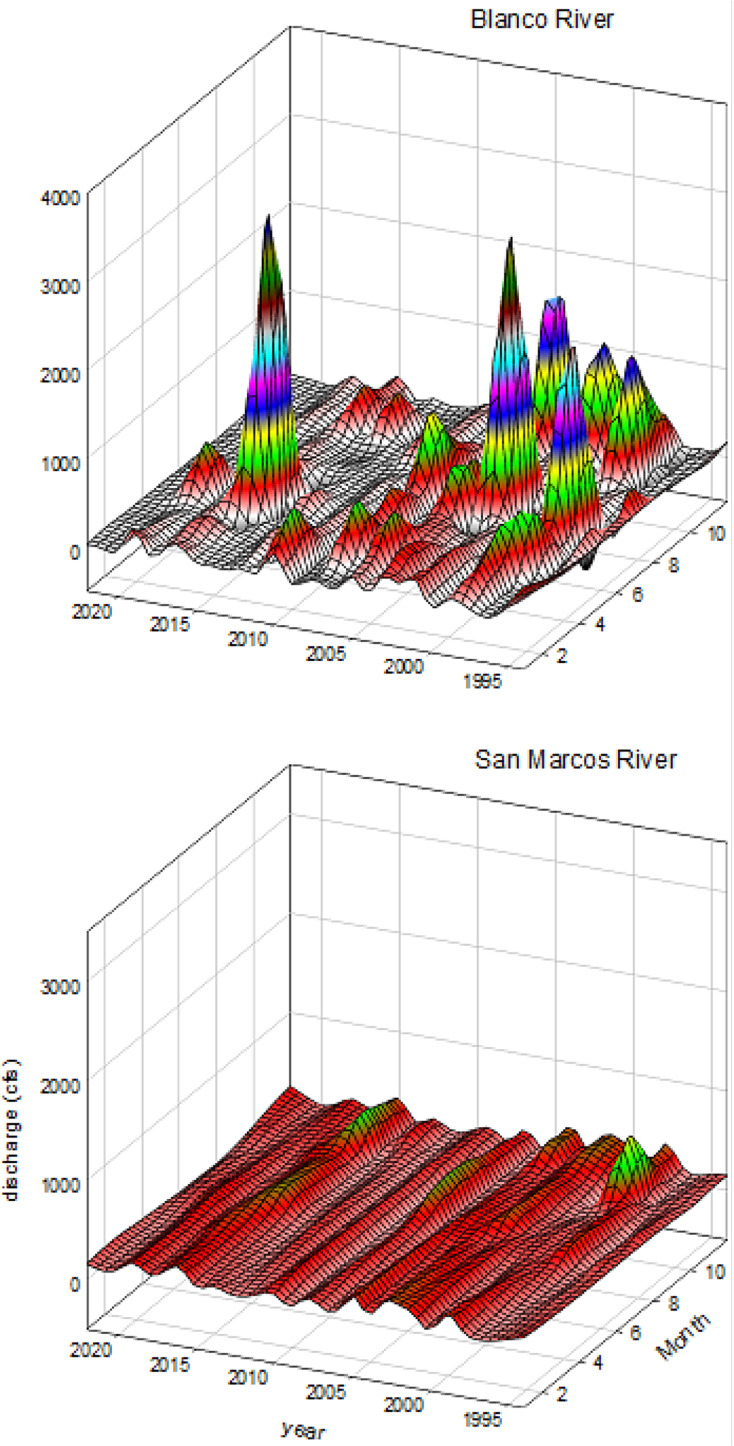
Mean monthly discharge (1993–2019) in hydrologically variable (Blanco River) and hydrologically stable (San Marcos River) systems. Three-dimensional surface plots of mean monthly discharge from 1993–2019. The top panel represents the hydrologically variable Blanco River, and the bottom panel represents the hydrologically stable San Marcos River.

**Table 1:** Summary of sampling sites depicted in Fig. 1.

River	Reach name	Site designation	Site name	Habitat type	Distance from spring outflows (rkm)	Latitude	Longitude
San Marcos River	Spring Lake	1	Spring Lake	NW	0	29.8939	−97.9303
		2	Spring Lake	NW	0	29.891	−97.9341
		3	Spring Lake	NW	0	29.8904	−97.9336
	Upper	4	Sewell Park	W	0.69	29.8893	−97.934
		5	Sewell Park	NW	0.81	29.8877	−97.9342
		6	Sewell Park	NW	0.9	29.8877	−97.9342
		7	Veterans Plaza	W	1.51	29.8827	−97.9351
		8	Rio Vista Park	NW	2.06	29.879	−97.9326
		9	Crooks Park	W	2.51	29.8762	−97.9322
		10	Olympic Outdoor Center	NW	2.89	29.8744	−97.9311
	Lower	11	Thompson Island	NW	4.22	29.869	−97.9288
		12	Water treatment plant	W	5.15	29.864	−97.9263
Comal River	Upper	13	Upper Spring Run	W	0	29.7204	−98.1279
		14	Upper Spring Run	NW	0	29.7204	−98.1279
		15	Upper Spring Run	NW	0	29.7155	−98.1331
		16	Upper Spring Run	NW	0	29.7168	−98.1325
	Landa Lake	17	Landa Lake	NW	0	29.7138	−98.1353
		18	Landa Lake	NW	0	29.7131	−98.1349
		19	Landa Lake	NW	0	29.7129	−98.1348
	New Channel	20	New Channel	NW	1.15	29.7069	−98.1289
		21	New Channel	NW	1.45	29.7077	−98.1276
		22	New Channel	NW	1.54	29.7077	−98.1265
		23	New Channel	NW	1.64	29.7076	−98.1247
	Old Channel	24	Old Channel	NW	2.28	29.7108	−98.1228
		25	Old Channel	NW	2.36	29.7106	−98.1225
		26	Old Channel	NW	2.41	29.7106	−98.1225
		27	Old Channel	W	2.52	29.7093	−98.1225

**Note:**

Includes river name, reach, site number, site name, habitat type (NW, non-wadeable; W, wadeable), distance from spring outflows (rkm), and geographic coordinates (latitude and longitude). rkm, river kilometer.

**Figure 2:**
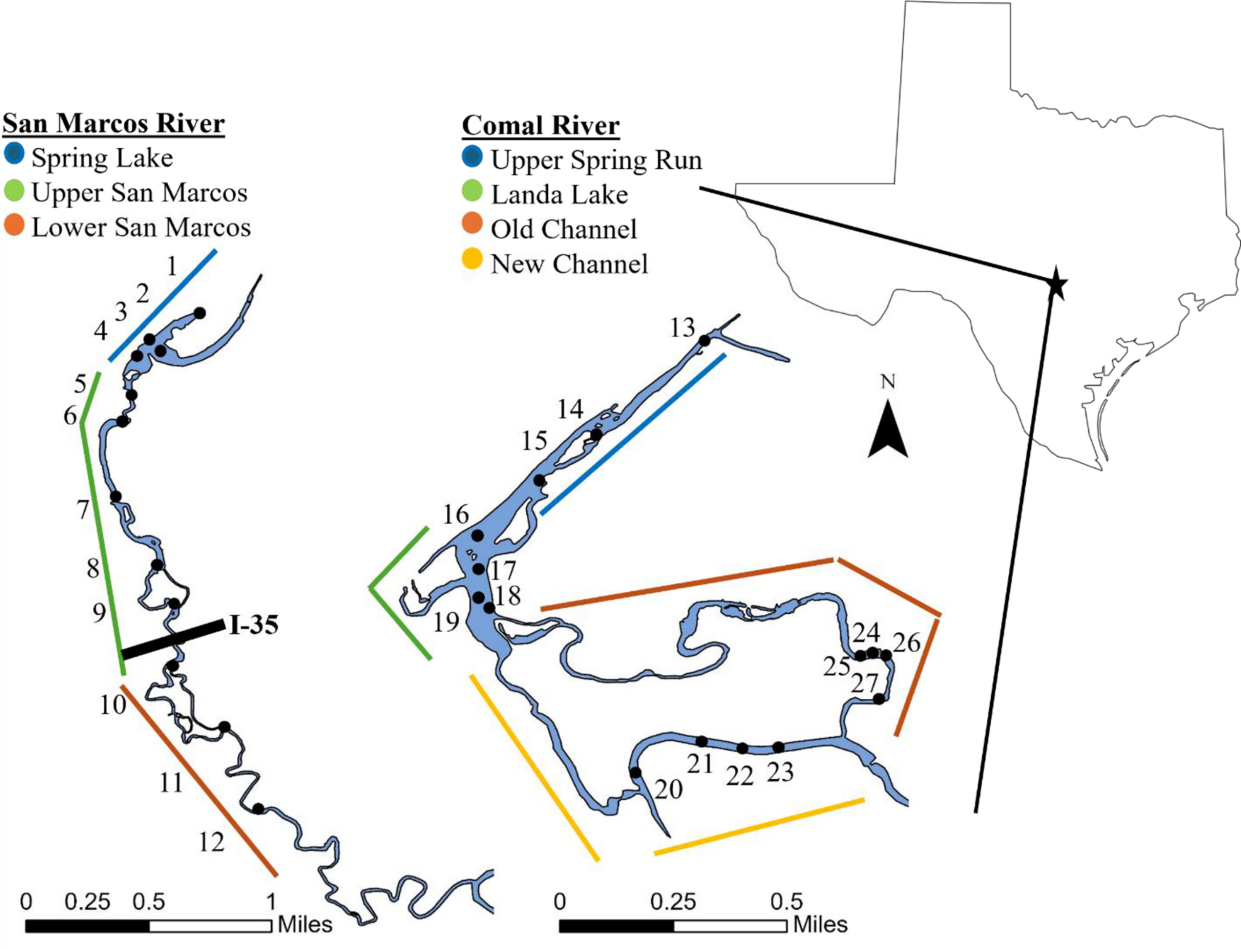
Map of the sample sites along the San Marcos River and Comal River, Texas, USA. Black circles indicate sampling sites (1–27). Colored lines represent designated reaches. Map created in ArcGIS Pro ESRI.

### Field surveys

Fish surveys were conducted in wadeable areas (<1.6 m in depth) with standardized seine hauls and in non-wadeable areas (>1.6 m in depth with some exceptions during low flow) with standardized visual surveys and SCUBA gear, which is commonly used in deep freshwater systems with high water clarity (Work & Jennings, 2019). The two sampling methodologies differed in areal coverage among sampling areas and in their ability to detect various species and sizes of individuals. Consequently, abundances from the two methodologies were not combined in subsequent analyses to avoid conflating sampling bias. Instead, two independent assessments were made with the fish survey data, one for wadeable areas and one for non-wadeable areas. In wadeable areas, fishes were quantified with a seine (3.0 × 1.8 m common sense seine; mesh size: 3.2 mm) at two sites in the upper San Marcos River and two sites in the lower San Marcos River and one site each in Upper Spring Run, Old Channel, and New Channel of the Comal River. Spring Lake in the San Marcos River and Landa Lake in the Comal River lacked sufficient amounts of wadeable areas for sampling with seines. Twenty seine hauls were targeted for each site and sampling event. Starting downstream and progressing upstream, a 5-m section of wadeable areas was sampled with a downstream haul in slower current velocity (<0.4 m/s) or the seine was set on the stream bottom and 5-m section in front of the seine was kicked in swifter current velocities (>0.4 m/s). Additional seine hauls were made in parallel with the previous seine haul and within the same cross-section with ample spacing between hauls for minimal disturbance of adjacent areas. Once the cross section was completed, a new cross section was sampled, located 20 m upstream until a total of 20 seine hauls were completed. Occasionally, fewer seine hauls were made because of the lack of wadeable areas at the site, specifically when flows were above median flow. Fishes were identified to species and released after each seine haul. The following variables were measured after each seine haul: water depth, current velocity (Hach FH 950 velocity meter), percent substrate (*i.e*., clay, silt, sand, gravel, cobble, boulder), percent amount of woody debris, percent detritus coverage, and percent vegetation (overall coverage and by taxa) following standardized procedures (Edwards & Bonner, 2022; Chappell et al., 2024). Macrophytes were places into categories based on height (*i.e*., tall or short; Edwards & Bonner, 2022), and growth type: rosette with circular leaf arrangements radiating from short stem, and caulescent with leaves on elongate stems protruding above substrate and/or above water and not radiating (Table S1; Schuyler, 1984; De Wilde et al., 2014). Three types of algae (filamentous, epiphytic, and detrital) were grouped together as “algae”. Bryophyte was retained as a distinct group, because it is the most frequently occurring vegetation (29% occurrence in wadeable areas, 37% occurrence in non-wadeable areas) in the Comal River and often associated with occurrences and abundances of the federally listed Fountain Darter (Edwards & Bonner, 2022). Vegetation taxa with percent coverage >5% were included in a category for analysis, whereas rare plants (<5% combined coverage) were excluded from analysis.

Fishes were quantified in non-wadeable areas at three sites in Spring Lake, three sites in upper San Marcos River, two sites in lower San Marcos River, and three sites in Upper Spring Run, Landa Lake, Old Channel, and New Channel of the Comal River. Non-wadeable reaches were sampled at two levels of resolution: water column surveys to quantify pelagic and surface-oriented fishes, and benthic surveys to quantify benthic fishes. Four divers surveyed each reach by swimming evenly spaced transects across the site and visually identifying and counting fishes in the water column. Dive lanes and fields of view were coordinated to avoid double-counting following standard underwater survey protocols (Brock, 1954; Schill & Griffith, 1984). Most fishes were identified to species; however, two co-occurring *Gambusia* species (*G. geiseri* and *G. affinis*) could not be reliably distinguished underwater and were therefore grouped as *Gambusia* sp. Juvenile and adult *Lepomis* also formed mixed-species shoals that were difficult to separate visually, especially for juveniles, so divers either identified individuals to species when possible or recorded them as *Lepomis* sp. Florida Bass (*Micropterus salmoides*) and Largemouth Bass (*M. nigricans*) exhibit extensive hybridization in Texas waters (Lutz-Carrillo et al., 2022), and individuals cannot be reliably distinguished *in situ*. Accordingly, all *Micropterus* observed were classified collectively as the Largemouth Bass complex.

After completing water-column surveys, divers conducted benthic surveys within four 10-m² plots established using randomly placed PVC markers. Working from downstream to upstream, each diver systematically searched substrates, lifting cobble and inspecting vegetation with flashlights to locate benthic fishes. These were identified to species when possible; however, *Etheostoma fonticola* and *E. lepidum* are difficult to distinguish when rapidly darting, so divers identified individuals either to species or as *Etheostoma* sp. when uncertain. For each benthic plot, divers recorded habitat variables including water depth, benthic and water-column velocity, percent substrate composition, percent detritus cover, percent vegetation cover, and vegetation taxa. Collections were taken according to state permits (SPR-0601-159), federal permits (TE236730), and Texas State University IACUC (1036-1102-32).

### Habitat guild assignments

Among all fishes observed within wadeable and non-wadeable areas, only the most abundant taxa (*i.e*., >4,500 individuals per taxonomic group across all sampling events) were selected for habitat guild assignments and subsequent analyses to increase the likelihood of using taxa captured during every sampling event and to eliminate the temporal variability associated with rarely occurring fishes. Selected fishes were assigned to surface, pelagic, and benthic habitat guilds based on quantified water column use associations or professional judgement (Frimpong & Angermeier, 2009: FishTraits). Among those assigned to the pelagic guild, fishes were divided further into two categories, pelagic-specialist and pelagic-generalist, with pelagic-specialist guild denoting species that are typically found only within spring systems of the Edwards Plateau (*i.e*., spring-associated fishes) and pelagic-generalist guild denoting species that are found within and outside of spring systems of the Edwards Plateau (*i.e*., riverine fishes; Hubbs, 1995; Craig et al., 2016). Surface guild consisted of G*ambusia geiseri* and *Gambusia affinis*, grouped together as *Gambusia* sp. Pelagic-specialist guild consisted of *Dionda nigrotaeniata*, *Astyanax argentatus*, and *Notropis amabilis*. Pelagic-generalist guild consisted of *Lepomis* sp. (*i.e*., *Lepomis auritus, Lepomis macrochirus, Lepomis miniatus, Lepomis aquilensis, Lepomis microlophus, Lepomis cyanellus, Lepomis gulosus*), *Micropterus* sp. (*i.e*., *Micropterus nigricans, Micropterus salmoides, Micropterus punctatus, Micropterus dolomieu* and hybrids), and *Herichthys cyanoguttatus*. Benthic guild consisted of *Etheostoma fonticola* and *Etheostoma* sp. (*E. fonticola* and *E. lepidum*).

### Extreme flow classification

**Figure 3:**
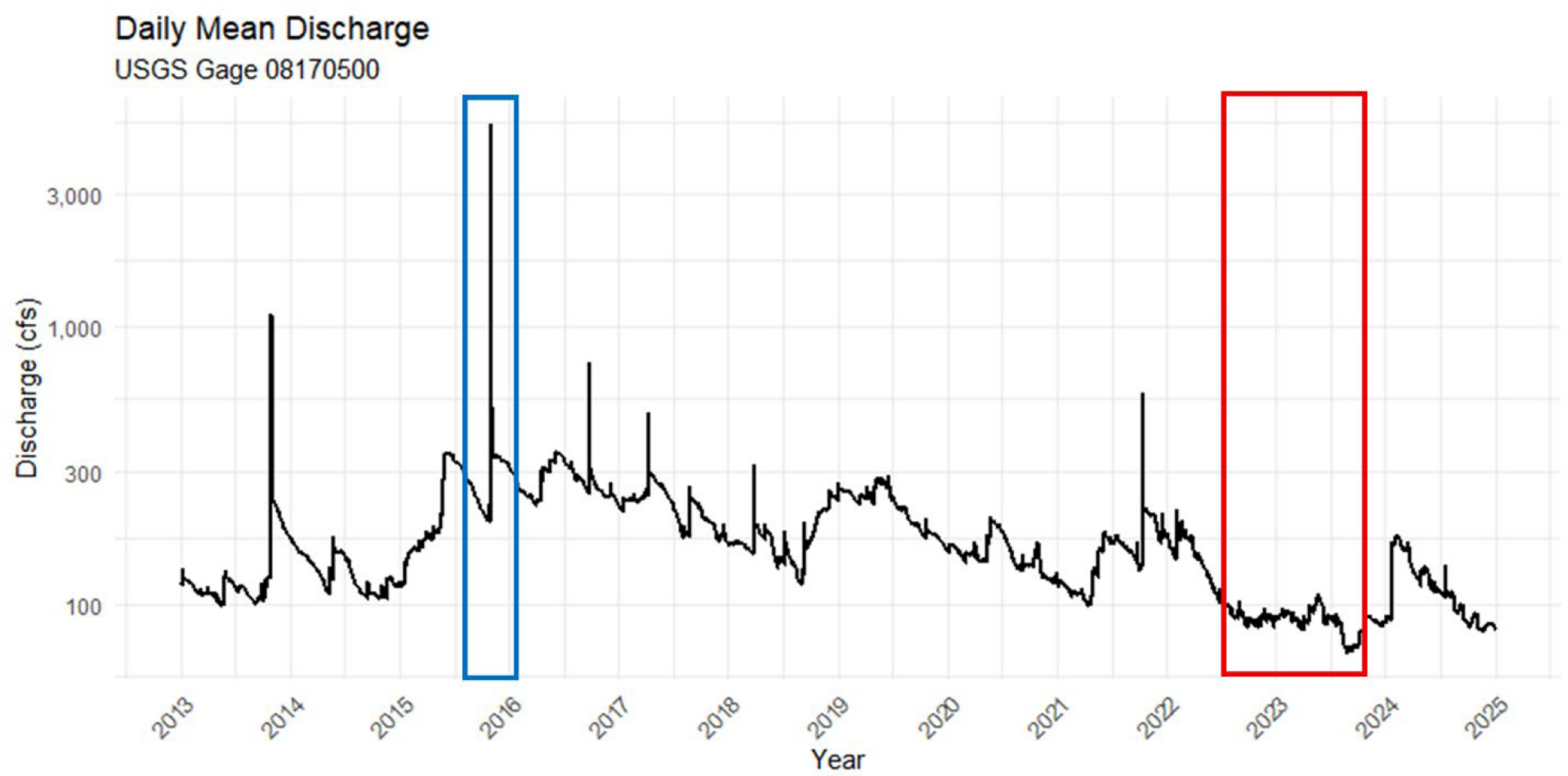
Hydrograph of San Marcos River at San Marcos, TX (USGS gage 08170500) discharge spanning 2013–2025. Blue outline indicates the flood period; red outline indicates the drought period.

During the 9-year period of this study, median flow was 4.87 m^3^/s (range: 2.19–527 m^3^/s) in the San Marcos River (USGS Station 08170500; D’Ottavio, 2020) and 8.50 m^3^/s (range: 2.18–400 m^3^/s) in the Comal River (USGS Station 08169000). Two extreme flow events (*i.e*., high flows during a major flood, low flows during severe or extreme droughts) were observed within both rivers (Fig. 3). Intensive precipitation fell within the San Marcos River and Comal River watersheds in October 2015, generating peak flows of 527 m^3^/s in the San Marcos River and 400 m^3^/s in the Comal River. The amount of precipitation caused a major flash flood, with overbanking flows and with an average frequency of >1 in 5 years, in both rivers based on flood classifications (Best, 2011) established at a downstream USGS Station (08172000) on the San Marcos River and a downstream USGS Station (08173900) on the Guadalupe River. Smaller high flow pulses (<68 m^3^/s in the San Marcos River, <60 m^3^/s in the Comal River) occurred during the study, but only the October 2015 flooding was considered an extreme high flow event in both rivers. Severe or extreme droughts occurred in both watersheds from June 2022 through November 2022, identified by the Standard Precipitation index (SPI; McKee, Doesken & Kliest, 1993). The median flow during severe and extreme drought was 2.6 and 3.23 m^3^/s in the San Marcos River and Comal River, respectively. Among the sampling events, one represented a major flood (*i.e*., first sampling event preceding flood conditions in all reaches), two sampling events in the San Marcos River and three sampling events in the Comal River represented drought, and the remaining sampling events represented stasis conditions (*i.e*., neither a high nor low flow event).

### Data analysis

A principal component analysis (PCA) was conducted to summarize habitat structure relationships. Centroids were calculated from PC axes 1 and 2 for each flow period, the sampling period in which the flow occurred (*i.e*., stasis, flood, drought), for wadeable and non-wadeable areas and were assessed for significance using a permutational Analysis of Variance (PERMANOVA). Vegetation coverage was assessed independent from other variables with a one factor ANOVA because it is a primary indicator of habitat structure, diversity, and abundance as well as habitat for species of interest in this study (Gorman & Karr, 1978; Rolls, Leigh & Sheldon, 2012; Edwards & Bonner, 2022).

Effects of extreme flows on fish were assessed using CPUE as a proxy for abundance separately for wadeable and non-wadeable areas using mixed effects models in the nlme package in RStudio (Pinheiro et al., 2017). For each species, the log-transformed CPUE [log(N+1)] was modeled as a function of flow period (*i.e*., stasis, flood, and drought) with a random intercept for reach to account for variability of repeated observations at the reach level. Temporal autocorrelation within reaches was accommodated using the autoregressive correlation structure corAR(1), which accounts for serial dependence in repeated observations over time (Pinheiro & Bates, 2000; Zuur et al., 2009). Because residual variance differed among flow periods, we applied a heterogeneous variance structure using VarIdent (Pinheiro & Bates, 2000). Estimated marginal means (EMMs) for flow periods were obtained from each model using emmeans. Pairwise differences among flow periods were evaluated on the model (log-transformed) scale using Tukey-adjusted comparisons. Pairwise contrast estimates, standard errors (SE), degrees of freedoms (df), t-values, and adjusted *p*-values were reported (Zuur et al., 2009). EMMs and confidence intervals were back-transformed for visualization of CPUE patterns at the taxa level.

## Results

A total of 335 sampling events (146 wadeable, 189 non-wadeable) occurred among the seven reaches of the San Marcos River and Comal River between May 2014 and November 2022. Across all reaches, wadeable areas consisted of swifter current velocities (range of means: 0.22–0.37 m/s), shallower depths (0.66–0.74 m) with predominantly gravel (32–37%) and silt (29–31%) substrates, and lesser vegetation coverage (37–39%); whereas, non-wadeable areas consisted of slower current velocities (0.1–0.2 m/s) and greater depths (1.6–1.7 m), with predominantly silt (39–64%) and gravel (14–25%) substrates and greater vegetation coverage (42–71%) (Table 2).

**Table 2:** Mean (± SE) physical habitat parameters across wadeable and non-wadeable areas for all sample types in the San Marcos and Comal rivers from May 2014–November 2022.

Reach	Wadeable		Non-wadeable	
River	San Marcos	Comal	San Marcos	Comal
Total observations (N)	1,452	1,296	807	1,311
Habitat parameters				
Current velocity (m/s)	0.37 (0.02)	0.22 (0.15)	0.2 (0.02)	0.1 (0.01)
Depth (m)	0.66 (0.02)	0.74 (0.18)	1.7 (0.18)	1.6 (0.02)
Substrate (%)				
Silt	31.2 (2.7)	28.9 (2.2)	33.8 (3.4)	63.6 (2.5)
Sand	15.1 (1.3)	10.2 (0.9)	19.1 (1.9)	2.6 (0.5)
Gravel	32.3 (1.8)	37.1 (1.6)	24.9 (2.5)	14.0 (1.6)
Cobble	13.6 (1.0)	13.2 (0.9)	10.2 (1.0)	4.1 (0.6)
Clay	2.3 (0.5)	0.3 (0.1)	10.1 (0.7)	2.7 (0.6)
Boulder	1.5 (0.2)	5.6 (0.8)	2.5 (0.2)	1.3 (0.3)
Bedrock	1.2 (0.4)	0.6 (0.2)	1.9 (0.2)	4.0 (1.0)
Vegetation cover (%)	36.8 (2.8)	38.7 (2.5)	41.6 (4.1)	71.3 (1.9)
Algae	1.5 (0.3)	10.3 (2.3)	7.4 (0.7)	12.3 (2.0)
Bryophyte	0.2 (0.1)	7.8 (1.3)	0.3 (0.03)	19.7 (1.8)
Short caulescent	4.5 (0.5)	12.1 (1.3)	5.3 (0.5)	16.6 (2.1)
Tall caulescent	13.4 (1.8)	3.5 (0.6)	18.9 (1.9)	7.3 (1.5)
Short rosette	0.2 (0.1)	0.7 (0.2)	1.7 (0.2)	4.5 (1.0)
Tall rosette	15.9 (1.7)	3.1 (0.9)	7.4 (0.7)	9.8 (1.8)

### Habitat response

**Figure 4:**
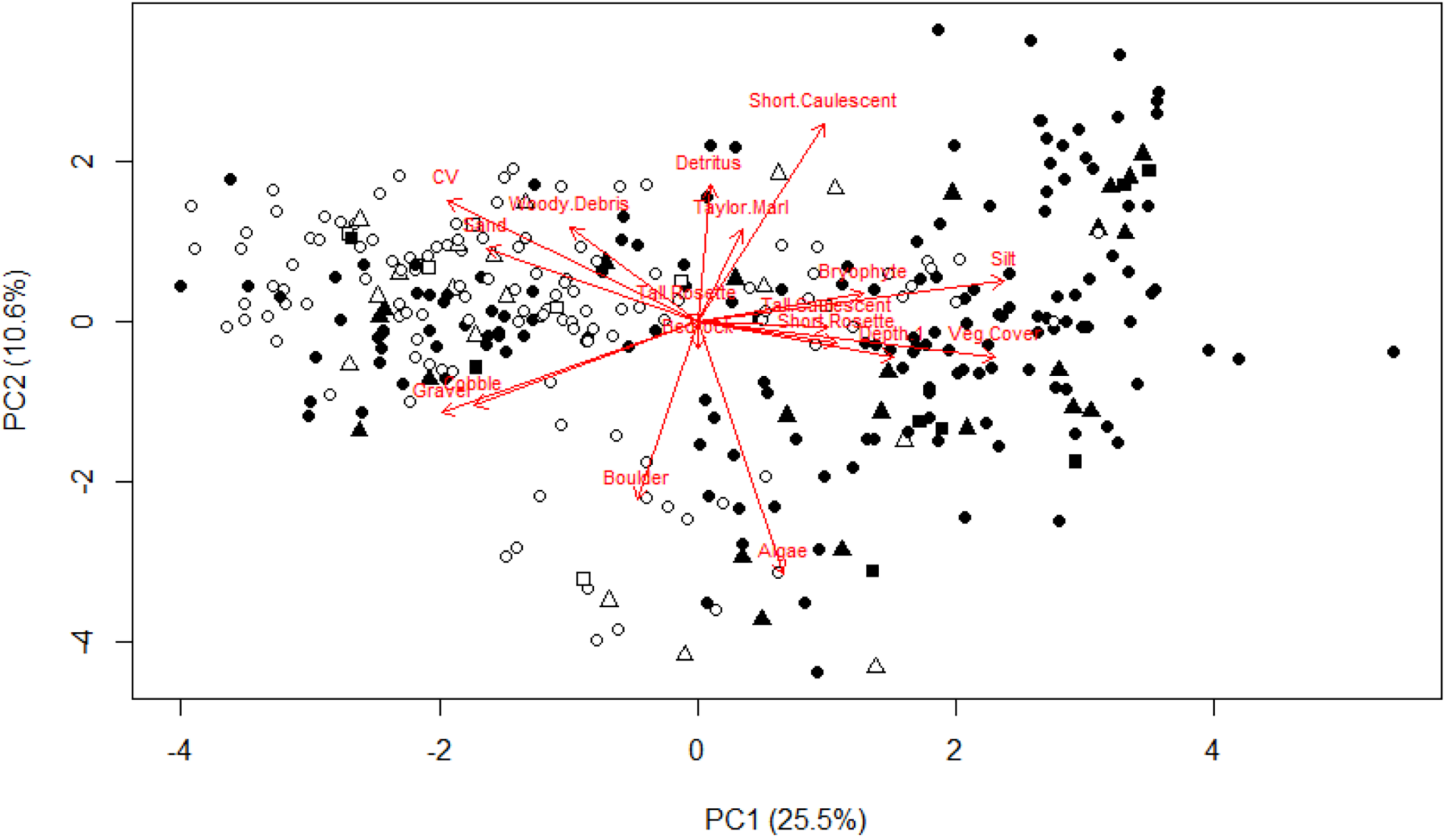
Principal component analysis (PCA) of habitat structure across sample events. Points represent individual sites classified by flow period (stasis = circle, flood = square, drought = triangle) and wadeability (non-wadeable = black, wadeable = white). Red arrows indicate loadings of environmental variables contributing to variation among sites. Percent variance explained by each axis is shown in parentheses.

Principal component axes I and II explained 36% of the variation in habitat variables taken among the 335 wadeable and non-wadeable areas. Principal component axis I explained 25% of the habitat variation and described a substrate, depth, and current velocity gradient. The strongest loadings on PC axis I were silt (−0.94), vegetation cover (−0.89), depth (−0.60), bryophyte (−0.44), sand (0.48), current velocity (0.55), cobble (0.63) and gravel (0.83). Principal component axis II explained 11% of the habitat variation and described a vegetation type, current velocity, and substrate gradient. The strongest loadings on PC axis II were tall rosette plants (−0.71), tall caulescent plants (−0.67), current velocity (−0.45), sand (−0.38), boulder (0.35), bryophyte (0.38), and algae (0.56). Mean (±1 SE) PC I scores by reach ranged from −1.36 (0.09) in non-wadeable areas of the Old Channel to 1.46 (0.08) in wadeable areas of lower San Marcos River, These scores indicate a gradient in habitat structure from swifter current velocity, heterogenous substrates, and low vegetation coverage in wadeable areas to slower current velocity, homogenous silt substrates, and increased vegetation cover in non-wadeable areas. The effects of flood and drought on habitat structure were not detected in wadeable or non-wadeable areas (PERMANOVA; *p* > 0.05) along PC axes 1 and 2 (Fig. 4). However, a one factor ANOVA displayed that flow period influenced vegetation coverage in wadeable areas (ANOVA, F_2,143_ = 3.17, *p* = 0.04) but not in non-wadeable areas (*p* = 0.3), where vegetation cover increased during drought (49% ± 8.19) in comparison to stasis (36% ± 4.56).

### Fish assemblage response

Forty-three species and 135,199 individuals were recorded from the San Marcos River and Comal River between May 2014 and November 2022. The most abundant taxa consisted of 17 species and 123,361 individuals, representing 91% of all individuals recorded. Non-wadeable areas accounted for 84% of all fish collected, dominated by pelagic specialists (39% in relative abundance), followed by surface (30%), pelagic generalists (18%), and the benthic guild (12%). Wadeable areas were dominated by surface fishes (65% in relative abundance), followed by pelagic generalists (15%), pelagic specialists (11%), and the benthic guild (8%).

In wadeable areas, effects of flow events were detected on pelagic generalist guild CPUE: *Lepomis* sp. and *H. cyanoguttatus* CPUE was lower (β = –0.0054, *p* = 0.03; β = –0.0008; *p* = 0.004, respectively) during flood than stasis, while *Micropterus* sp. CPUE was greater (β = 0.0108, *p* = 0.03) during drought than the flood period. Likewise, flow period effects were detected on pelagic specialist guild CPUE: *D. nigrotaeniata* CPUE was greater during drought (β = 0.0060, *p* = 0.0004; β = 0.0080; *p* = 0.012) relative to flood and stasis, and *A. argentatus* CPUE was lower (β = –0.007; *p* = 0.0001) during flood than stasis and was greater (β = 0.010; *p* = 0.0022) during drought than flood. Benthic guild CPUE was greater (β = 0.0124; *p* = 0.05) during drought than flood. Effects of flow events were not detected (*p* ≥ 0.25) on surface guild CPUE (*Gambusia* sp.) (Table 3). Overall, results suggest that in wadeable areas, flooding had a greater effect on decreasing CPUE and drought increased CPUE.

**Table 3:** Tukey-adjusted pairwise comparisons of log transformed CPUE among flow periods (drought, flood, stasis) for eight fish species in wadeable areas.

Species	Contrast	Estimate (β)	SE	df	t-value	*p*-value
*Lepomis* sp.	Drought–Flood	0.012	0.0057	139	2.156	0.08
	Drought–Stasis	0.0068	0.0055	139	1.254	0.42
	Flood–Stasis	−0.0054	0.0021	139	−2.538	**0.03**
*H. cyanoguttatus*	Drought–Flood	0.0013	0.0007	139	1.979	0.12
	Drought–Stasis	0.0005	0.0007	139	0.768	0.72
	Flood–Stasis	−0.0008	0.0002	139	−3.270	**0.004**
*Micropterus* sp.	Drought–Flood	0.0108	0.0042	139	2.553	**0.03**
	Drought–Stasis	0.0071	0.0041	139	1.722	0.20
	Flood–Stasis	−0.0037	0.0016	139	−2.273	0.06
*D. nigrotaeniata*	Drought–Flood	0.0080	0.0026	139	2.897	**0.012**
	Drought–Stasis	0.0060	0.0015	139	3.949	**0.0004**
	Flood–Stasis	−0.0016	0.0023	139	−0.720	0.75
*A. argentatus*	Drought–Flood	0.010	0.0029	139	3.446	**0.0022**
	Drought–Stasis	0.004	0.0032	139	1.118	0.50
	Flood–Stasis	−0.007	0.0015	139	−4.348	**0.0001**
*N. amabilis*	Drought–Flood	0.0109	0.0073	139	1.488	0.30
	Drought–Stasis	0.0026	0.0042	139	0.611	0.81
	Flood–Stasis	−0.0083	0.0064	139	−1.305	0.40
*E. fonticola*	Drought–Flood	0.0124	0.0052	139	2.404	**0.05**
	Drought–Stasis	0.0085	0.0039	139	2.157	0.08
	Flood–Stasis	−0.0039	0.0036	139	−1.104	0.51
*Gambusia* sp.	Drought–Flood	0.049	0.0310	139	1.590	0.25
	Drought–Stasis	0.009	0.0097	139	0.930	0.62
	Flood–Stasis	−0.040	0.0310	139	−1.295	0.40

**Note:**

Estimates (β), standard errors (SE), degrees of freedom (df), t‐values, and adjusted *p*‐values are shown. Bold *p*-values indicate significance.

**Figure 5:**
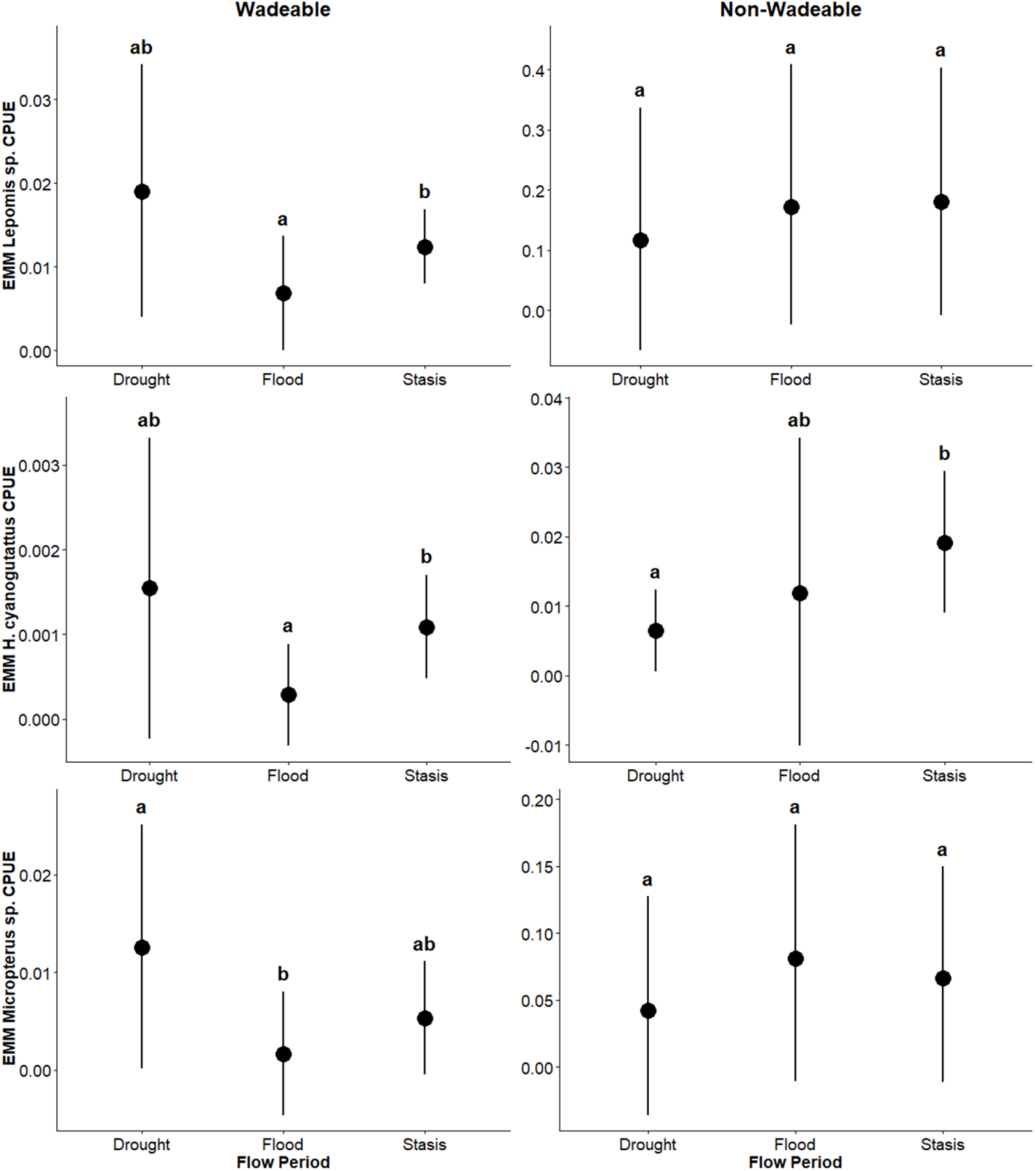
Estimated marginal means (EMMs ± 95% CI) of pelagic generalist guild density across flow periods (drought, flood, stasis) in wadeable (left) and non-wadeable (right) areas. Letters denote significant pairwise differences among flow periods within each habitat type (*p* < 0.05).

**Figure 6:**
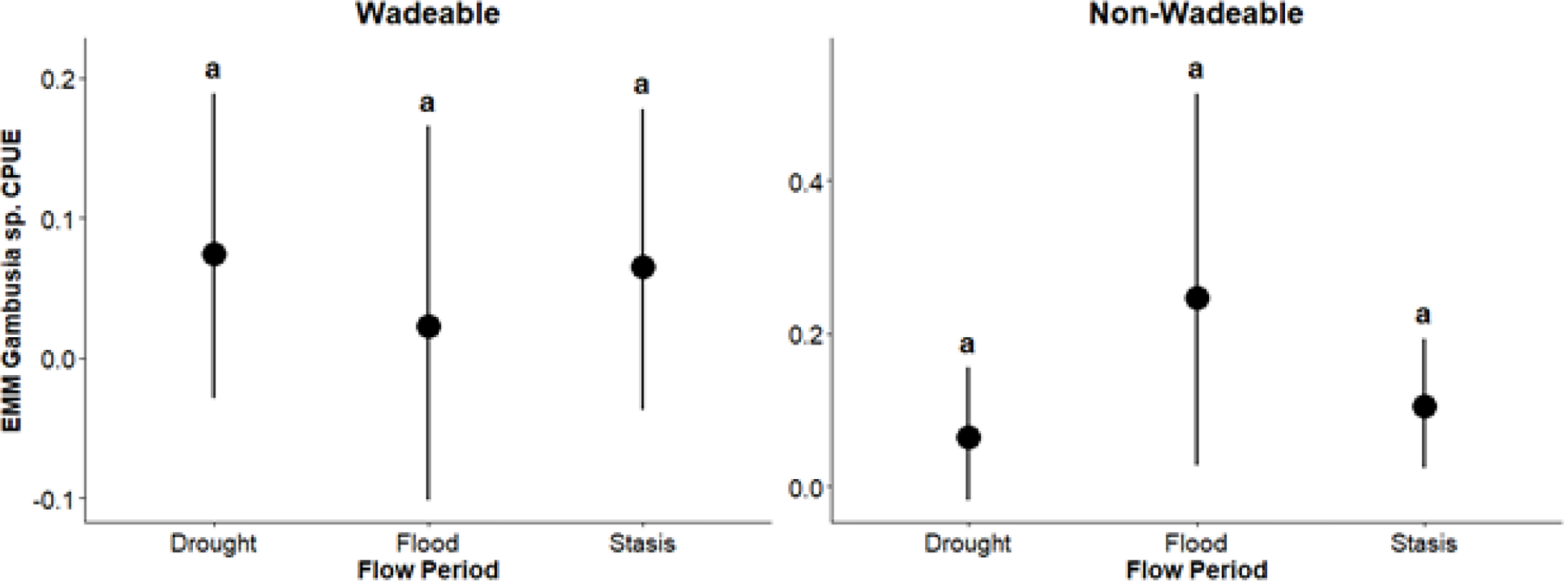
Estimated marginal means (EMMs ± 95% CI) of surface guild density across flow periods (drought, flood, stasis) in wadeable (left) and non-wadeable (right) areas. Letters denote significant pairwise differences among flow periods within each habitat type (*p* < 0.05).

**Figure 7:**
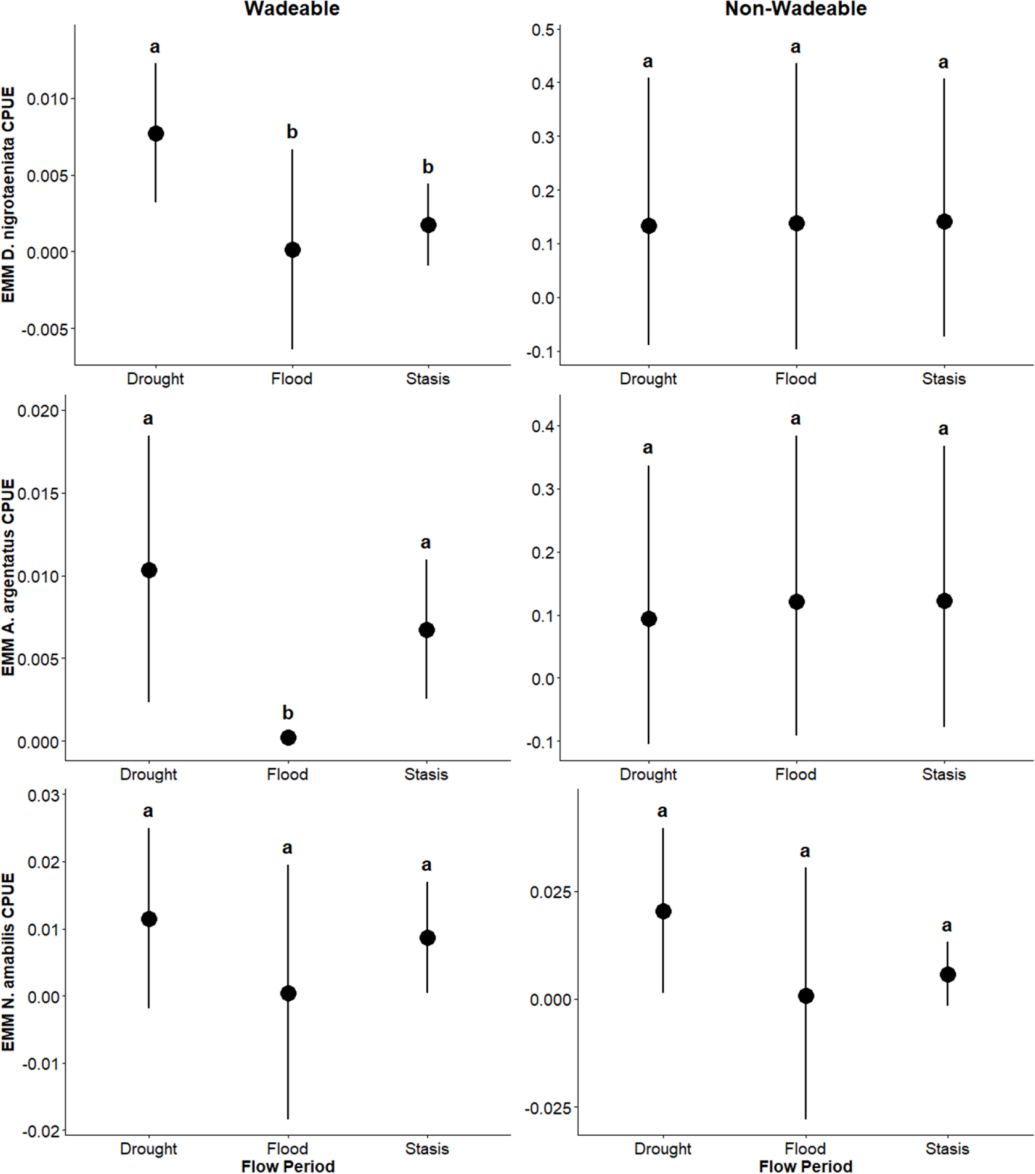
Estimated marginal means (EMMs ± 95% CI) of pelagic specialist guild density across flow periods (drought, flood, stasis) in wadeable (left) and non-wadeable (right) areas. Letters denote significant pairwise differences among flow periods within each habitat type (*p* < 0.05).

**Figure 8:**
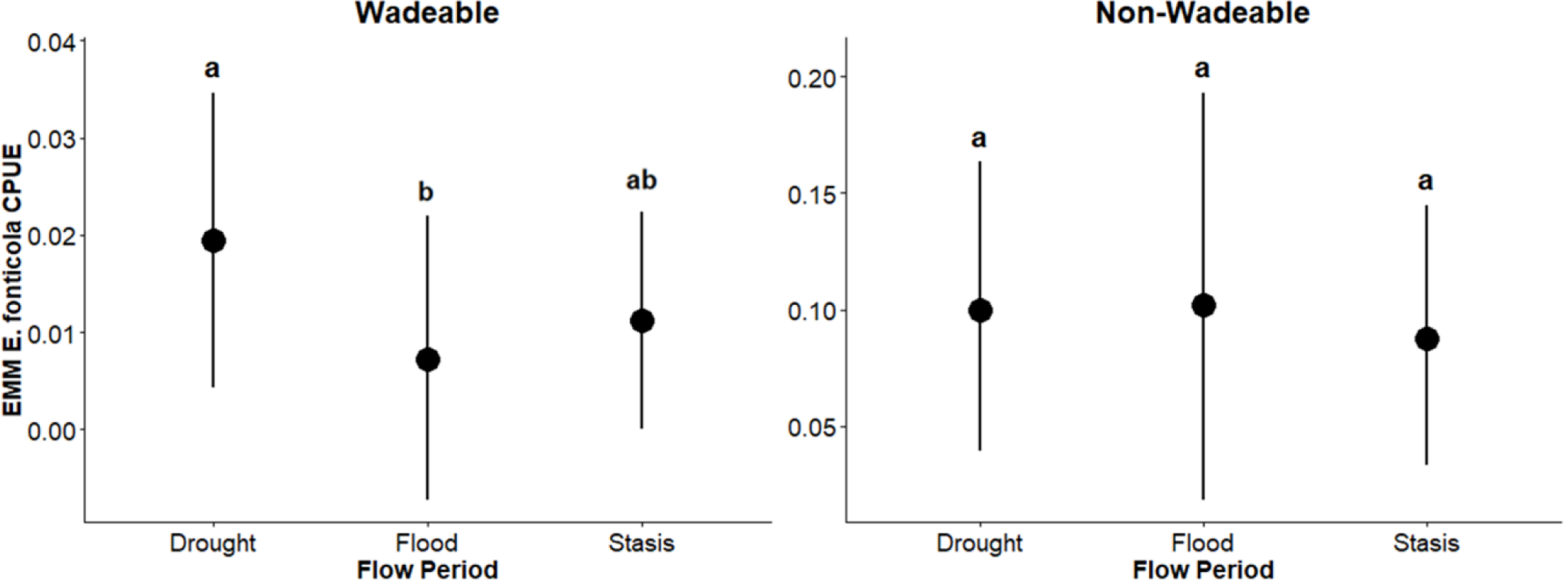
Estimated marginal means (EMMs ± 95% CI) of benthic guild density across flow periods (drought, flood, stasis) in wadeable (left) and non-wadeable (right) areas. Letters denote significant pairwise differences among flow periods within each habitat type (*p* < 0.05).

In non-wadeable areas, effects of flow events were not detected (*p* ≥ 0.20) on pelagic generalist guild CPUE for *Lepomis* sp. and *Micropterus* sp. (Fig 5). For pelagic generalist *H. cyanoguttatus*, CPUE was lower (β = –0.013; *p* = 0.004) during drought events than stasis events (Fig. 5). Effects of flow events were not detected (*p* ≥ 0.18) on surface guild CPUE (*Gambusia* sp.; Fig. 6), pelagic specialist guild CPUE (*D. nigrotaeniata, A. argentatus, N. amabilis*; Fig. 7), and benthic guild CPUE (*E. fonticola* and *Etheostoma* sp.; Fig. 8, Table 4). Collectively, results suggest that fishes in deeper, non-wadeable areas were resistant to short term hydrological events.

**Table 4:** Tukey‐adjusted pairwise comparisons of log CPUE among flow periods (drought, flood, stasis) for eight fish species in non-wadeable areas.

Species	Contrast	Estimate (β)	SE	df	t-value	*p*-value
*Lepomis* sp.	Drought–Flood	−0.095	0.060	180	−1.579	0.26
	Drought–Stasis	−0.023	0.023	180	−0.724	0.75
	Flood–Stasis	0.072	0.058	180	1.252	0.42
*H. cyanoguttatus*	Drought–Flood	−0.0065	0.009	180	−0.709	0.76
	Drought–Stasis	−0.013	0.004	180	−3.251	**0.004**
	Flood–Stasis	−0.0063	0.010	180	−0.650	0.79
*Micropterus* sp.	Drought–Flood	−0.050	0.032	180	−1.558	0.27
	Drought–Stasis	−0.017	0.012	180	−1.342	0.37
	Flood–Stasis	0.034	0.032	180	1.059	0.54
*D. nigrotaeniata*	Drought–Flood	−0.012	0.082	180	−0.147	0.98
	Drought–Stasis	0.029	0.033	180	0.972	0.66
	Flood–Stasis	−0.041	0.080	180	0.511	0.86
*A. argentatus*	Drought–Flood	−0.019	0.067	180	−0.286	0.96
	Drought–Stasis	−0.012	0.028	180	−0.421	0.91
	Flood–Stasis	0.007	0.066	180	0.113	0.99
*N. amabilis*	Drought–Flood	0.019	0.014	180	1.372	0.36
	Drought–Stasis	0.014	0.008	180	1.782	0.18
	Flood–Stasis	−0.005	0.012	180	−0.401	0.92
*E. fonticola*	Drought–Flood	0.014	0.031	180	0.444	0.90
	Drought–Stasis	0.016	0.015	180	1.047	0.55
	Flood–Stasis	0.002	0.029	180	0.066	0.99
*Gambusia* sp.	Drought–Flood	−0.13	0.088	180	−1.530	0.28
	Drought–Stasis	−0.036	0.021	180	−1.730	0.20
	Flood–Stasis	0.098	0.087	180	1.127	0.50

**Note:**

Estimates (β), standard errors (SE), degrees of freedom (df), t‐values, and adjusted *p*‐values are shown. Bold *p*-values indicate significance.

## Discussion

The premise of our initial predictions is that the effects of extreme flow periods would be more pronounced in hydrologically stable systems than in hydrologically variable systems. Among wadeable areas, study results supported some of our initial predictions, including a habitat change during drought (*i.e*., increases in algae) and decreases in two pelagic generalist taxa following flood event. One unexpected result was the decrease in a pelagic specialist (*i.e*., *A. argentatus*) abundance following a flood event. Other notable results were the increase in abundance of benthic guild fishes during drought and the lack of detectable effects on benthic guild fish abundance following a flood event. Among non-wadeable areas, study results did not support our initial predictions (*e.g*., reduced vegetation, decreases in pelagic generalist abundances) during extreme flow events, with the only exception of a decrease in abundance of *H. cyanoguttatus* during drought. Collectively, we concluded that the effects of extreme flow periods on habitat structure and fish assemblages in hydrologically stable systems were similar to, or unexpectedly less pronounce, than the effects of flow periods on hydrologically variable systems.

Fish assemblage—flow period relationships were limited in non-wadeable areas with only *H. cyanoguttatus* (pelagic generalist) having lower CPUE in drought relative to stasis conditions. The lack of assemblage—flow period responses is likely attributed to the habitat stability, consistent baseflows, and stable water quality within groundwater-fed systems (Davis et al., 2013), and specifically the San Marcos and Comal rivers (Groeger et al., 1997; Saunders et al., 2001; BIO-WEST, Inc., 2024). In hydrologically variable systems, fishes typically disperse into or are trapped within deeper, refuge habitats during drought as surrounding areas dry or become disconnected, thereby fluctuating the density of non-wadeable habitats (Magoulick & Kobza, 2003; Magoulick et al., 2021).

Within wadeable areas, pelagic generalists declined in CPUE during floods, likely attributed to their deep, laterally compressed morphology that is unfavorable for sustained swimming (Webb, 1984) and is more suited for lentic environments (Moyle & Cech, 2004). This can cause individuals to be displaced downstream, particularly in areas with limited flood refuge (Perkin & Gido, 2011). Unexpectedly, *A. argentatus* (pelagic specialist guild) displayed lower CPUE during the flood period relative to stasis conditions. It is possible that flooding conditions exceeded the critical swimming speed of *A. argentatus* (~51 cm/s; Leavy & Bonner, 2009; Katopodis & Gervais, 2016); however, since this CPUE decrease was not detected for *D. nigrotaeniata* (~18 cm/s), another explanation for lower CPUE could be resource availability and acquisition. *A. argentatus* is an invertivore-drift predator that relies on sight-feeding (Estrada, 1999); therefore, increased turbidity associated with flood pulses can influence species dispersal into deeper channels and/or off-channel refugia where prey capture ability increases (Whitty et al., 2009), inevitably decreasing individual capture likelihood (Junk, Bayley & Sparks, 1989; Bayley, 1995). During drought conditions in wadeable areas, members from multiple guilds demonstrated positive CPUE—flow period relationships. Specifically, *Micropterus* sp. (pelagic generalist), *D. nigrotaeniata* (pelagic specialist), and *Etheostoma* (benthic) increased CPUE during the drought period. Drought conditions are known to cause a concentration effect due to habitat loss and fish movement to refugia (Magoulick & Kobza, 2003). While this concentration of individuals is common in hydrologically variable systems where drought causes stream disconnectivity and reduces available niche space (Matthews & Marsh-Matthews, 2003), the hydrologically stable systems of this study are perennial and did not display stream drying or longitudinal disconnectivity. However, during drought current velocities decreased in some wadeable areas, potentially leading to better capture success of large pelagic fishes as lower water depths made escape difficult. The increase in CPUE of the benthic guild during drought was unexpected, as historical studies identified *E. fonticola* to be susceptible to habitat alteration and extreme low flows (Hubbs & Peden, 1969; Schenck & Whiteside, 1976). Recent studies into the vegetation associations of *E. fonticola* identified high correlations of individuals with bryophyte and other similar benthic macrophytes (*e.g*., algae; Edwards & Bonner, 2022). *D. nigrotaeniata* is also a vegetation associated species, primarily feeding on algae, detritus, and small invertebrates (Hubbs & Garrett, 1990); therefore, increases in CPUE during drought correlate with the detected increase in vegetation cover.

Effects of drought on fish assemblages are more pronounced in hydrologically variable systems, where decreased species richness and increased species mortality are attributed to the loss of lateral, longitudinal, and vertical surface water connectivity and stream drying (Pringle, 2001; Larned et al., 2010; Magoulick et al., 2021), extreme warmer water temperatures, and dissolved oxygen variability (Davies, 1978; Caruso, 2002; Van Vliet & Zwolsman, 2008). Within our study reaches, groundwater outflows from San Marcos and Comal springs were sufficient to maintain stream connectivity and water quality during drought thereby mitigating effects typically observed in hydrologically variable systems and providing an explicit illustration on how flows from these spring systems are decoupled from local climates (Davis et al., 2013; Craig et al., 2016). However, flows from these spring systems during drought are not guaranteed or infinite, especially when combined with groundwater pumping from the same aquifers (Schenck & Whiteside, 1976; Craig & Bonner, 2021). Also in this study, responses of some species (*i.e*., *Gambusia*) to flood differed from those in an arid region stream, where surface fishes, including *Gambusia*, decreased by >75% in relative abundances (Meffe, 1984). Collectively, fish responses in this study are similar to responses in other hydrologically stable systems where flow stability is associated with water quality and fish assemblage stability, despite occasional extremes in flow caused by unpredictable flooding or drought (Poff & Allan, 1995; Lynch, Leasure & Magoulick, 2018; Magoulick et al., 2021).

Anthropogenic modifications (*e.g*., channel dredging, non-native introductions, low head dam construction) have been occurring within our study systems since the 1850’s (Kollaus et al., 2015; Thiels, Edwards & Bonner, 2022) and thus could limit the inference when relating to other hydrologically stable systems. Low head dams specifically, but also channel dredging, artificially increase water depth and hydraulic retention (Turgeon, Turpin & Gregory-Eaves, 2019) and, therefore, can potentially increase habitat structure and flow refugia used by aquatic species (Hitt & Angermeier, 2008). Historical accounts, prior to anthropogenic flow modifications, described the San Marcos as a deep narrow stream and the Comal River as having a large swamp downstream from spring outflows (Brune, 2002). As a result, we believe that anthropogenic modifications to the natural flow regime have led to more dynamic spring systems where riffle/pool structure and heterogenous habitat resemble other natural spring system dynamics (Brune, 2002; Kollaus et al., 2015).

Past approaches into understanding the effects of flow extremes on fish assemblage and habitat have been primarily focused on intermittent/runoff streams where only one type of flow extreme is present within the study (Ross & Baker, 1983; Pearsons, Li & Lamberti, 1992; Bunn & Arthington, 2002; Willis, Winemiller & Lopez-Fernandez, 2004; Huang et al., 2025), with fewer studies analyzing impacts of both events on hydrologically stable systems (but see Schlechte & Fleming, 2015; Magoulick et al., 2021). Previous studies have also focused primarily on threatened and endangered species (Schenck & Whiteside, 1976; Schenck & Whiteside, 1977; Bonner et al., 1998; Dwyer et al., 2005; Edwards & Bonner, 2022; Poole et al., 2022), without identifying assemblage level responses to extreme flows. This study provides novel insights into guild-level patterns of species density in relation to habitat structure and extreme flow events. By examining guild composition across flow periods, we observed that assemblage—flow period relationships vary by habitat, likely due to resource and habitat availability, consistent baseflows, and stable water quality that align with species ecological requirements (Saunders et al., 2001; Perkin et al., 2015; Chappell et al., 2024). Pronounced decreases in CPUE were also identified, potentially relating to a lack of high flow tolerance or limited reproductive success (Lytle & Poff, 2004). By assessing species that encompass multiple habitat usage guilds, we provide a more holistic perspective and framework for understanding how groundwater dominated fish assemblages change under flow extremes.

Historically, these hydrologically stable systems have been thought to be sensitive to environmental change in the forms of habitat alteration (Hubbs & Peden, 1969) and extreme flow events (Schenck & Whiteside, 1976). Based on the results of this study, hydrologically stable systems offer greater resistance to flow extremes than previously thought, despite flow extreme levels in this study being less than those reported in the past (max flood levels near 2,265 m^3^/s, min flow levels reaching 0.16 m^3^/s). While extreme flow events are an integral mechanism in the organization of aquatic habitat structure and for maintaining prominent levels of biodiversity (Pearsons, Li & Lamberti, 1992; Lake, 2000; Poff, 2018; Rolls, Leigh & Sheldon, 2012), the effects are different for hydrologically variable and stable systems. Hydrologically stable systems offer dynamically structured habitats that are crucial for maintaining high taxa densities that are resistant, within both the habitat and community, to alterations from extreme flow events.

## Conclusions

Our findings demonstrate that hydrologically stable spring-fed rivers resist major changes in habitat during extreme flows, but fish assemblages respond in variable ways, with pelagic generalists and specialists showing declines under floods or droughts, while *Micropterus* and *Etheostoma fonticola* increased during drought. These results highlight both the resilience and vulnerability of spring systems: although groundwater buffering reduces habitat alteration, biotic responses reveal potential risks under future climate scenarios. Future research should integrate long-term monitoring of guild-specific responses with experimental approaches to disentangle the roles of habitat structure, competition, and dispersal in shaping resilience. As climate change intensifies extreme flow events and groundwater extraction pressures increase, assessing the thresholds of resilience and resistance in spring systems will be critical for guiding conservation of endemic and imperiled fishes.

## Supplemental Information

10.7717/peerj.21092/supp-1 Supplemental Information 1 Aquatic vegetation taxa identified by height and growth form among reaches in the San Marcos River and Comal River. Common (> 5% cover) aquatic vegetation taxa identified by height (tall or short) and growth form (Bryophyte, algae, caulescent and rosette) among wadeable and non-wadeable reaches in the San Marcos River and Comal River from May 2014—November 2022.

## Additional Information and Declarations

### Data Availability

The following information was supplied regarding data availability:

The data is available at Texas Data Repository: Tivin, Joshua; Bonner, Timothy, 2023, “Biological monitoring master dataset”, https://doi.org/10.18738/T8/M1KOP9, Texas Data Repository, V1, UNF:6:Ec1Qik+z+KdPkf4BqWM74A== [fileUNF].
