# Supplementary material for: Influence of extreme flows on habitat and fish assemblage structure in groundwater-dominated systems
Source: PeerJ. 2026 Apr 27;14:e21092. doi: 10.7717/peerj.21092 (PMC13131351; doi:10.7717/peerj.21092)
Supplement: Supplemental Information 1 — Common (> 5% cover) aquatic vegetation taxa identified by height (tall or short) and growth form (Bryophyte, algae, caulescent and rosette) among wadeable and non-wadeable reaches in the San Marcos River and Comal River from May 2014—November 2022. [file peerj-14-21092-s001.docx]

| **Lowest Taxonomic Level** | **Common Name** | **Height** | **Classification** | |
| --- | --- | --- | --- | --- |
| *Bryophyta* | Bryophyte | Short | | Bryophyte |
| Detrital Algae | Detrital Algae | Short | | Algae |
| Filamentous Algae | Filamentous Algae | Short | | Algae |
| Epiphytic Algae | Epiphytic Algae | Short | | Algae |
| *Hydrilla verticillata* | Hydrilla | Tall | | Caulescent |
| *Cabomba caroliniana* | Cabomba | Tall | | Caulescent |
| *Characeae* | Chara | Tall | | Caulescent |
| *Haloragaceae* | Myrio-Millfoil & parrotfeather | Tall | | Caulescent |
| *Ceratophylum demersum* | coontail | Tall | | Caulescent |
| *Justicia americana* | Justicia | Tall | | Caulescent |
| Potamogetonaceae | Potamogeton | Tall | | Caulescent |
| *Hygrophila lacustris* | Hygrophila | Short | | Caulescent |
| *Ludwigia repens* | Ludwigia | Short | | Caulescent |
| *Hydrocotyle verticillata* | Hyrdocotyle pennywart | Short | | Caulescent |
| *Vallsinaria americana* | Vallsineria | Tall | | Rosette |
| *Zizania texanus* | Texas Wild Rice | Tall | | Rosette |
| *Sagittaria platyphylla* | Sagitarria | Short | | Rosette |

**Supplementary table 1.** Common (> 5% cover) aquatic vegetation taxa identified by height (tall or short) and growth form (Bryophyte, algae, caulescent and rosette) among wadeable and non-wadeable reaches in the San Marcos River and Comal River from May 2014 - November 2022.
